# Estimating lake ice thickness in Central Ontario

**DOI:** 10.1371/journal.pone.0208519

**Published:** 2018-12-06

**Authors:** Justin C. Murfitt, Laura C. Brown, Stephen E. L. Howell

**Affiliations:** 1 Department of Geography, University of Toronto Mississauga, Mississauga, Ontario, Canada; 2 Climate Research Division, Environment and Climate Change Canada, Toronto, Ontario, Canada; Northeastern University, UNITED STATES

## Abstract

Lakes are a key geographical feature in Canada and have an impact on the regional climate. In the winter, they are important for recreational activities such as snowmobiling and ice fishing and act as part of an important supply route for northern communities. The ability to accurately report lake ice characteristics such as thickness is vital, however, it is underreported in Canada and there is a lack of lake ice thickness records for temperate latitude areas such as Central Ontario. Here, we evaluate the application of previously developed temperature models and RADARSAT-2 for estimating lake ice thickness in Central Ontario and provide insight into the regions long term ice thickness variability. The ALS Environmental Science Shallow Water Ice Profiler (SWIP) was used for validation of both temperature and radar-based models. Results indicate that the traditional approach that uses temperatures to predict ice thickness during ice growth has low RMSE values of 2.3 cm and correlations of greater than 0.9. For ice decay, similar low RMSE values of 2.1 cm and high correlations of 0.97 were found. Using RADARSAT-2 to estimate ice thickness results in R^2^ values of 0.6 (p < 0.01) but high RMSE values of 11.7 cm. Uncertainty in the RADARSAT-2 approach may be linked to unexplored questions about scattering mechanisms and the interaction of radar signal with mid-latitude lake ice. The application of optimized temperature models to a long-term temperature record revealed a thinning of ice cover by 0.81 cm per decade.

## Introduction

Lakes are an important feature of the Canadian landscape and hold 7% of the world’s freshwater resources [1]. The presence of lakes can have an impact on the surrounding climate, particularly the impact noted on the net radiation balance and latent heat flux [2]. The presence of ice-free lakes in winter months can contribute to lake-effect snow and understanding the impact of lakes on climate is important for climate models [2,3]. Lake ice also acts as an important indicator of how regional climate is changing. Lake ice coverage has been connected to recent increases in temperature, with shifts in the date of freeze up by 10.7 days later and 8.8 days earlier for ice off compared to mean dates from a 150 year record [4]. Changes in temperature can also impact economic and recreational activities in areas that rely on lake ice. Recent research on the Tibbitt to Contwoyto Winter Road in Canada’s Northwest Territories has shown that increasing temperatures are reducing lake ice thickness along the key economical route and impacting the number of days in operation [5]. Additionally, there is increasing interest in lake ice due to the impact on under-ice biological communities [6]. Although lake ice plays an important role in local economies, there is a paucity in lake ice thickness monitoring [7].

Lake ice thickness has historically been estimated using a combination of non-linear and linear temperature models (e.g. [8–10]). Stefan’s equation [8] is the oldest model for estimating ice thickness and uses thermal conductivity, latent heat of fusion of ice, and ice density to calculate thickness [11]. However, this data may not be readily available for certain locations and more recent models have adapted the model to use primarily the non-linear relationship between ice thickness and accumulated freezing degree days (AFDD). These models are also adjusted based on different surface types and conditions [9,12,13]. The rate of ice decay and ice thickness during melt can be represented using linear models, with temperature represented by accumulated thawing degree days (ATDD) [10,14]. Although AFDD and ATDD methods have proven successful in northern latitudes, they have had limited application in mid-latitudes and the application of these models has focused on river ice (e.g. [15]). Therefore, research is needed to determine if the models remain applicable at lower latitudes.

In order to estimate ice thickness using the temperature-based model, climate data is needed from local weather stations, unfortunately, the distribution of temperature stations is sparse throughout regions of Canada and is not representative for estimating ice thickness across large areas. Satellite remote sensing provides an alternative to these temperature-based methods and can cover large spatial areas, allowing for ice thickness measurements at the regional scale. Passive microwave satellite observations have been used to estimate ice thickness (e.g. [16,17]), however, the coarse spatial resolution of these satellites (typically 25 km) limits its utility for small lakes. Synthetic aperture radar (SAR) systems (such as RADARSAT-2 and the European Remote Sensing Satellites (ERS) 1/2) have a finer spatial resolution (less than 100 m) and are able to resolve lakes with a smaller surface area [18,19] and have been used in combination with visible imagery to predict ice thickness and depth of shallow lakes [20,21]. In addition, research has demonstrated strong correlations between SAR observations and modelled ice thickness [18]. Similar to ice estimates from temperature, remote sensing techniques to estimate freshwater lake ice thickness have seen limited application in mid-latitudes. Therefore, it is unclear whether or not these methods will see similar success for lake ice in these regions where ice conditions differ from the northern latitudes [22,23].

This study addresses the paucity of in situ lake ice thickness records in the mid-latitudes through testing the applicability of both temperature based and remote sensing based ice thickness models. Specifically, the objectives of this study were to; i) estimate lake ice thickness in Central Ontario using established temperature models, ii) determine the applicability of using SAR from RADARSAT-2 for estimating ice thickness in Central Ontario, and iii) investigate long-term trends in ice thickness for Central Ontario.

## Methods

### Study site

**Fig 1:**
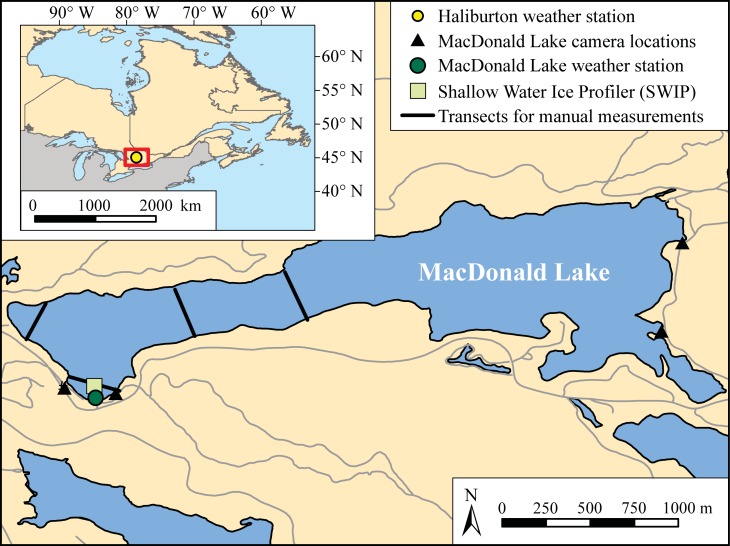
MacDonald Lake in the Haliburton Forest and Wildlife Reserve Ltd. Property, the dark green dot indicates the location of the on-shore weather tower; the light green square is the location of the Shallow Water Ice Profiler, placed less than 30 metres off-shore in view of the weather tower; the black triangles show the location of shoreline cameras used for observing daily ice conditions; and the black lines represent the transects where manual ice thickness measurements were collected. The location of the Haliburton weather station (yellow circle), which is the location for the homogenized long-term temperature data, is shown.

The focus of this study is MacDonald Lake, located in the Haliburton Forest and Wildlife Reserve Limited near the town of Haliburton, Ontario, Canada, which is situated in the Canadian Shield and dominated by temperate forests (Fig 1). Permission was granted by the owners of the Haliburton Forest and Wildlife Reserve Limited to conduct field work on the property. The lake is ~1.6 km^2^ with a maximum depth of 39.6 m [24] and was selected to represent a typical small/medium sized lake in the region. Climatology from the nearest government weather station (Haliburton 3) indicates a temperature range of -9.9°C in January to 18.7°C in August, with 1073.5 mm of annual precipitation (26% falling as snow) [25]. The Haliburton Highlands and Algonquin regions of Central Ontario rely heavily on lake ice in the winter season for tourism as over 100,000 visitors came to the regions for snowmobiling in 2016 [26]. Ice cover typically forms between mid-December to early January and the process of melt begins between the middle of March and the beginning of April. Full ice cover typically lasts until the end of April but can persist until early May [22,27].

### Temperature data

Air temperature data for MacDonald Lake were acquired using a weather station that was constructed on-shore in the fall of 2015 (Fig 1). The tower collects data on several climate variables including humidity, precipitation, snow depth, and temperature, averaged or sampled on the hour. In order to expand the time frame investigated, supplementary daily mean temperature records from the homogenized Canadian surface air temperature dataset were used (data available at https://open.canada.ca/data/en/dataset/d6813de6-b20a-46cc-8990-01862ae15c5f) [28,29]. These data are available for Environment and Climate Change Canada’s Haliburton weather station (78.53° W, 45.03° N) and span June 1888 to December 2017. Six ice seasons had to be removed from the analysis due to missing temperature data, 1931–1932, 1932–1933, 1933–1934, 1937–1938, 1968–1969, and 2006–2007.

### Ice thickness

To obtain a consistent record of lake ice measurements throughout the 2016–2017 ice season, an ALS Environmental Science Shallow Water Ice Profiler (SWIP) was deployed before ice formation in August 2016 (Fig 1). The SWIP uses acoustic sounding in order to measure the return range from the bottom of the ice surface to the profiler [30,31]. This range is used in combination with temperature, pressure, and sensor orientation to calculate ice thickness. In addition to data from the SWIP, manual measurements were taken on MacDonald Lake during stable ice cover in both 2015–2016 and 2016–2017 ice seasons. These data were collected from four transects distributed across MacDonald Lake to provide a representation of ice conditions across the lake (Fig 1). These data were averaged to provide a single ice thickness for each collection date. Shoreline observations of ice conditions were also recorded during the 2015–2016 and 2016–2017 ice seasons by a series of 4 *Reconyx Hyperfire* cameras positioned on the trees around MacDonald Lake and provided an hourly record of surface conditions during daylight hours (06:00 to 18:00 EST) (Fig 1).

### Ice thickness estimates from temperature

Lebedev [9] proposed an equation to estimate sea ice thickness, simplifying Stefan’s ice thickness law for sea ice [8], using AFDD, which are calculated once temperatures are consistently below 0° C, and a modifier that depends on the environmental conditions of the site. Eq (1), based on the equation proposed by [9] for estimating sea ice thickness, is as follows:

$$h_{i}=\beta_{F}*{AFDD}^{\gamma }$$ (1)

where *h*_*i*_ is the ice thickness, AFDD is accumulated freezing degree days and *β*_*F*_ and *γ* are constants that reflect the influence of the environment and average snow conditions on the rate of ice growth. Note that the subscript *F* was added here to differentiate *β*_*F*_ from *β*_*T*_ in the melt equation [10]. The equation has been used at various sites to determine the thickness of sea ice (eg. [10,12]) and is used by the United States Army Corps of Engineers (USACE) for calculating river ice thickness [15]. When the equation is applied to a lake with average snow cover, values for *β*_*F*_ range from 1.7–2.4 when a constant value of 0.5 is used for *γ* [13,15]. Due to the availability of shoreline temperature data and SWIP data, the 2016–2017 ice season was used to develop the models for Central Ontario and these available data were split into two groups, 67% (n = 76) used to train the initial model and 33% (n = 38) used to validate the model. Using the non-linear least squares (nls) function in R [32,33], two equations were tested, one using an optimized value for *β*_*F*_ and *γ* = 0.5 [12,15], the other used an optimized value for *β*_*F*_ and *γ*. Both equations were determined for these 2016–2017 data by evaluating the relationship between AFDD and ice thickness measurements collected by the SWIP.

To estimate ice thickness during the process of ice decay, linear regression was used. Bilello [10] developed an equation that uses ATDD to estimate the rate of sea ice decay (2):

h=0.55*ATDD (2)

where *h* is the decrease in ice thickness (cm / ATDD above -1.8°C) and ATDD is the accumulated thawing degree days. The equation was shown to have a correlation coefficient of 0.93 [10]. However, this equation only determines the rate of decay, not ice thickness. In order to determine ice thickness during decay, a modified equation from Bilello [14] includes a term that represents maximum ice thickness (3):

$$h_{i}=I_{m}-\beta_{T}*ATDD$$ (3)

where *h*_*i*_ is ice thickness, *I*_*m*_ is maximum ice thickness, *β*_*T*_, is the rate of ice decay (cm/ ATDD) between the date of maximum ice thickness and the date of water clear of ice (note: subscript *T* was used here to differentiate the *β*_*F*_ in the melt equation), and ATDD is the accumulated thawing degree days [14]. During an analysis of ATDD and the timing of ice off for MacDonald Lake, it was determined that 90 ATDD was an appropriate empirical cut-off for when ice had completely melted (see section 3.1.2 for further details). This was used in combination with the maximum ice thickness determined from Eq (1) to calculate a slope that represented the rate of ice decay in Central Ontario for each year. This slope was unique for each year, 2009–2017, and was used to determine the daily ice thickness during melt.

The root mean square error (RMSE) and Kendall’s Tau-b were calculated in order to quantify the accuracy of both non-linear and linear models [34–39]. Additionally, Willmott’s refined index of agreement was calculated between the SWIP measured ice thickness and the temperature-based thickness estimates [40]. The index of agreement provides the sum of the error-magnitudes relative to the sum perfect-model-deviation and observed-deviation magnitudes [40]. Comparisons were also made between the temperature-estimated ice thickness and the manually collected measurements during both the 2015–2016 and 2016–2017 ice seasons. Eqs (1) and (3) were also applied to the full temperature dataset ranging from 1888–2017 to evaluate long-term trends in ice thickness for Central Ontario. Trends in the long-term dataset were calculated using the “Zhang” method of trend analysis [41,42]. The Zhang method is based on Sen’s slope and limits the influence of autocorrelation within the time series using an iterative process to accurately calculate the trend coefficients [41–43].

### Ice thickness estimates from RADARSAT-2

In order to evaluate the applicability of radar in estimating ice thickness, 382 HH polarized RADARSAT-2 ScanSAR wide images were acquired for Central Ontario from 2008 to 2017. Due to data sharing agreements currently in place for the recent RADARSAT data the backscatter return values for this data cannot be distributed, though the data is available from MDA (https://mdacorporation.com/) through fee-based usage. Due to constraints on data availability, gaps between images generally ranged from 4–7 days. The images were processed using ESA’s Sentinel Application Platform (SNAP) [44]. SNAP was used to radiometrically correct the images from digital numbers (DN) into usable measurements of backscatter (sigma nought values) in decibels (dB) [44,45]. Images were geometrically corrected so that images would match real world features using the SRTM 1 second grid DEM, which was recorded by the space shuttle Endeavour from February 11–22, 2000 [44,46,47]. The backscatter in these images was normalised to an incidence angle of 39° by adjusting the backscatter by 0.26 dB/°. This was done by modifying a backscatter normalisation for sea ice [48] to be applicable for lakes in mid-latitudes (see [22] for full explanation of the processing and normalisation methods). Using a 150-metre shoreline buffered lake mask, the mean backscatter value for MacDonald Lake was extracted from each available image.

To estimate ice thickness using RADARSAT-2 data, a linear regression model was established for the 2016–2017 ice season using mean backscatter and ice thickness from the SWIP measurements. These data were split into training and validation sets, with 68% (n = 30) as training and 32% (n = 14) as validation. The model results were validated using Kendall’s Tau-b [34–39] correlations between data from the SWIP and HH estimated ice thickness as well as Willmott’s refined index of agreement [40].

## Results

### Estimating ice thickness using temperature

#### Ice growth

**Fig 2:**
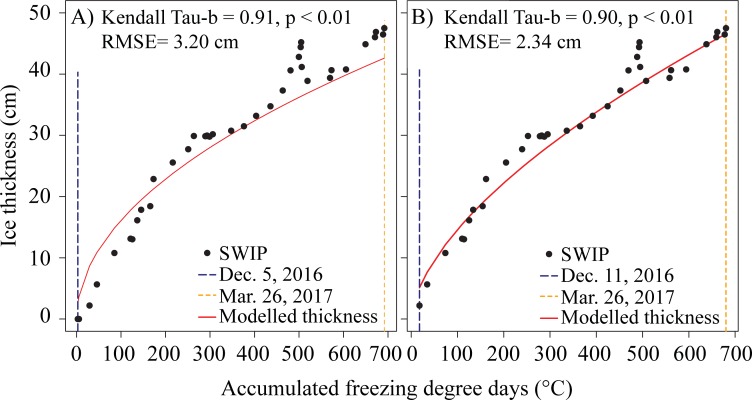
Comparison of the initial and optimized AFDD model. The initial model (A) (optimized *β*_F_ of 1.73 and constant *γ* of 0.5) on the validation dataset for the 2016–2017 ice season, the dark blue solid line is December 5^th^, the date of initial thickening, and the orange solid line is March 26^th,^ the date of maximum ice thickness. The optimized AFDD model (B) (optimized *β*_F_ of 0.94 and optimized *γ* of 0.60) on the validation dataset for the 2016–2017 ice season. Ice thickening begins December 11^th^, 2016 (blue line) and maximum ice thickness is reached on March 26^th^, 2017 (orange line). The solid circles represent the daily average ice thickness from the SWIP.

Fig 2 illustrates a strong relationship between temperature and predicted ice thickness with a correlation of 0.91 and an RMSE of 3.2 cm. Although the regression works well to predict ice thickness later in the season, it initially overpredicts the ice thickness when the AFDD are below ~200°C (Fig 2A). Additionally, there is a grouping of points when the AFDD are between 481 to 495°C (February 16^th^ to February 26^th^) where there is a spike in thickness values, however, this is not captured by the model (Fig 2A). Based on the MacDonald Lake weather station data, the mean daily temperatures on Feb 16^th^ and 17^th^ were -11°C, however, the temperatures on Feb 16^th^ into the morning of Feb 17^th^ were very low (reaching -22°C by 8:00 am on Feb 17^th^) and resulted in the rapid increase in ice thickness seen in the data from the SWIP. By the afternoon of Feb 17^th^ temperatures rose above 0°C, followed by several days where melt was observed, before returning to freezing conditions by Feb. 26^th^, after which the ice resumed thickening. Daily mean temperatures were used to tally the AFDD, so the large diurnal variation during this brief cold snap and subsequent melt was not captured by the model. After this event, the curve continued to represent the thickness well for the remainder of the season.

The second model developed used the modified Eq (1) that optimized both *β*_F_ and *γ* as done in previous literature for estimating sea ice thickness in the Russian Arctic [9]. Additionally, it was found in the first model that the AFDD resulted in an earlier freeze up date in 2016 compared to shoreline images (December 4^th^ verses December 10^th^), to more accurately represent shoreline observations the model was forced to start on the later date (December 10^th^). Fig 2B illustrates that the optimized model, with a *β*_F_ value of 0.94 and an increase in the value of *γ* to 0.60, resulted in a slightly lower correlation of 0.90 but an improved RMSE of 2.3 cm (Fig 2B). Furthermore, the RMSE remains comparable between the training and validation data, 2.28 cm and 2.34 cm respectively. The curve cannot capture the late February spike in ice thickness, similar to the original model, and overestimates the ice thickness for the points leading to maximum thickness. However, optimizing both *β*_F_ and *γ* results in a better overall fit to the validation data as there is less overestimation of ice thickness at the beginning of the ice season (Fig 2B). Based on the improved RMSE values and similar correlation statistics, the second model was selected for use in further analysis and will be referred to as the ‘optimized AFDD model’ in the subsequent text.

The optimized AFDD model also accurately represents mean ice thickness determined from the manual measurements collected at the auger holes (Table 1). Focussing on the 2016–2017 season, ice thickness was successfully estimated on January 20^th^, February 3^rd^, and February 17^th^ with differences ranging from 0.41 to 3.24 (Table 1). Although a slightly larger difference (3.79 cm) was observed on February 10^th^, the estimate is still reasonable and is mostly likely a result of the SWIP influence on the degree day estimates, as the SWIP also reports thicker ice. The lower estimate on February 21^st^ is most likely due to the outlier in manual ice thickness measurements (58.5 cm) resulting in the wide range of values that day (± 6.4 cm) (Table 1). Differences between the field reported ice thickness and degree day estimates were also small in 2016, ranging from 2.40 to 5.53 cm (Table 1), showing that the model can be useful for other years when no SWIP data are available.

**Table 1:** Comparison between SWIP, temperature estimated ice thickness, and field reported ice thickness for available dates in 2016 and 2017 (in centimetres). Each field reported ice thickness value also includes the standard deviation for the measurement.

Date	SWIP (cm)	Degree Day Ice Thickness Estimate (cm)	Field Reported Ice Thickness (cm)	Difference between Degree Day Estimates and Field Reported Thickness (cm)
**2016**				
Feb. 12		32.38	28.25 ± 0.25	4.13
Feb. 16		36.53	31.53 ± 2.28	5.00
Feb. 26		40.48	34.95 ± 2.00	5.53
Mar. 4		43.97	41.57 ± 5.64	2.40
**2017**				
Jan. 20	29.87	28.37	28.78 ± 3.19	0.41
Feb. 03	31.48	33.02	29.78 ± 2.06	3.24
Feb. 10	35.78	36.84	33.05 ± 2.30	3.79
Feb. 17	44.40	39.57	40.45 ± 3.58	0.88
Feb. 21	44.58	39.69	44.38 ± 6.43	4.69

**Fig 3:**
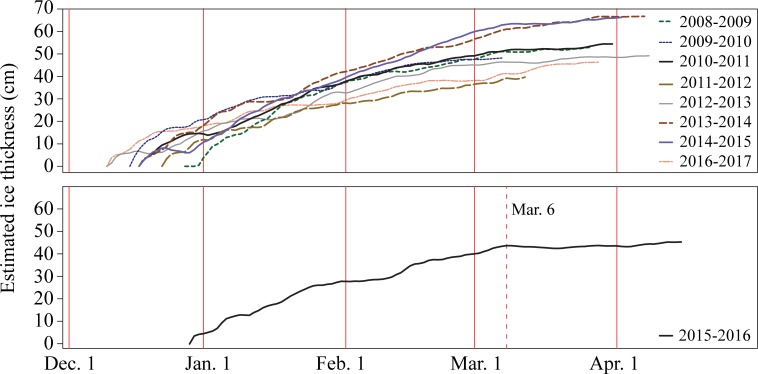
Yearly ice growth curves for the 2008–2009 to 2014–2015, and 2016–2017 ice seasons (top). Ice growth curve for the 2015–2016 ice season (bottom), which was separated to show the double AFDD peak, where the dotted red line represents the first AFDD maximum that was used to estimate ice thickness in the optimized model. The solid red lines represent the first of each month as indicated.

In order to test the applicability of the optimized AFDD model to previous years of data, ice growth curves from other ice seasons were compared to the 2016–2017 curve. The start date for the optimized AFDD model for the seasons prior to camera installation was determined using RADARSAT-2 imagery (see [22] for full methodology), while the 2015–2016 season start date was determined from the shoreline cameras. The growth of ice thickness from 2008–2009 to 2014–2015 follows a similar curve to what is seen using these 2016–2017 data (Fig 3). Although ice growth during the 2015–2016 ice season follows a similar pattern to the other ice seasons, towards the end of the season, two AFDD peaks can be found (Fig 3). The first AFDD peak, with a value of 602°C, is reported on March 6^th^ and corresponds with shoreline images that show melt onset starting on March 7^th^. However, on April 13^th^ there is a higher peak in AFDD with a value of 639.46°C. This is due to a period of colder temperatures, and visible refreeze with a minimum mean daily temperature of -8.4°C and maximum daily means that do not exceed 5.3°C. Due to the lack of SWIP data this year and unsafe ice condition preventing field data collection, we are unsure of the behaviour of ice growth during this time, though it is likely that ice thickness may have increased during this cold snap in the melt season.

#### Ice decay

In order to determine the rate of ice decay during melt, an empirical cut-off needs to be set using ATDD. During the 2016–2017 ice season, ATDD reached 88.8°C by the end of the melt season, which coincides well with other years that were investigated where ATDD values generally reached 90°C by the water clear of ice date observed in MODIS visible imagery. Water clear of ice dates that were determined using the 90 ATDD cut-off were found to be 0 to 5 days different from the dates visible in satellite imagery and shoreline data for 2009–2017 ice seasons. Therefore, to calculate ice thickness using Eq (3), the rate of ice decay between maximum ice thickness from the optimized AFDD model estimates and water clear of ice was calculated for all ice seasons using a value of 90 as the maximum ATDD (i.e. to represent the date when water clear of ice is reached). This model is referred to as the ‘ATDD model’ henceforth.

**Fig 4:**
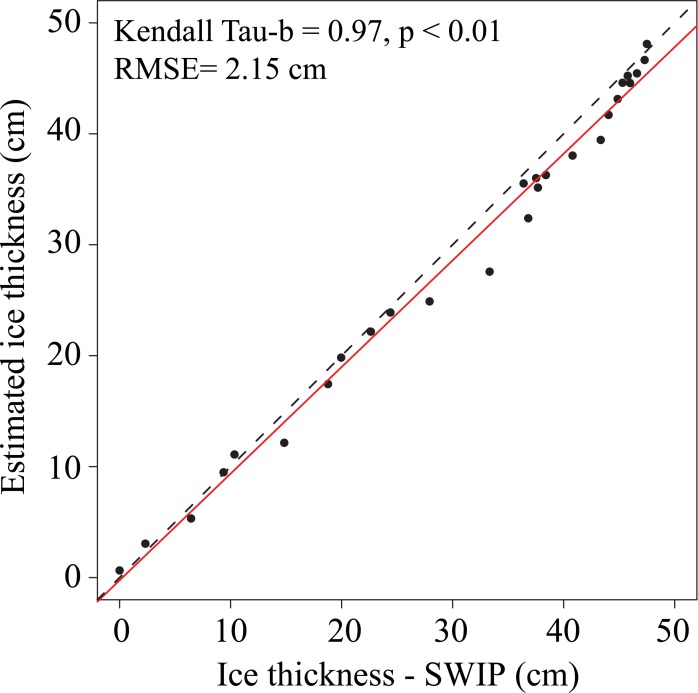
Comparison between ice thickness estimates based on the optimized ATDD model and the SWIP measurements for the 2016–2017 ice season.

The method was initially tested on 2016–2017 data and compared to the ice thickness measurements obtained by the SWIP. Using the maximum ice thickness determined by the optimized AFDD model, the slope calculated for ATDD and ice thickness had a value of -0.53 cm/ATDD. When plotting these estimated ice thickness data against SWIP measurements, a linear relationship was found with a slope of 0.96, a Kendall tau-b of 0.97 (p < 0.01), and an RMSE of 2.15 cm (Fig 4).

For the 2009–2016 ice seasons, slopes range from -0.44 to -0.74 cm/ATDD (Table 2). Higher rates of decay are connected to higher maximum AFDD, as the larger maximum ice thickness values result in steeper slopes between maximum thickness and 90 ATDD. The slowest rate of decay was found in 2012 when ice was the thinnest and the fastest rates of decay were found in 2014 and 2015 when ice was the thickest (Table 2). The faster rates of decay in 2014 and 2015 were due to cooler temperatures in March and followed by warmer temperatures near the end of April and beginning of May.

**Table 2:** Maximum estimated ice thickness and rate of ice decay for 2009–2016.

Year	Estimated Maximum Ice Thickness (cm)	Maximum AFDD (°C)	Rate of Ice Decay (cm/ATDD)	Water clear of ice date (WCI)	March (°C)	April (°C)	May (°C)
2009	52.96	798.10	-0.59	Apr. 24	-2.92	4.18	10.26
2010	48.20	682.60	-0.54	Apr. 4	1.33	7.78	13.98
2011	54.44	835.20	-0.60	Apr. 30	-4.15	4.28	12.96
2012	39.68	494.60	-0.44	Mar. 26	2.51	4.57	14.10
2013	49.22	706.70	-0.55	Apr. 28	-2.51	3.44	12.43
2014	66.75	1170.90	-0.74	May 5	-8.78	2.77	12.15
2015	66.27	1157.10	-0.74	Apr. 28	-5.57	3.95	13.68
2016	44.68	602.00	-0.50	Apr. 25	-2.15	1.10	11.82
2017	48.10	680.35	-0.53	Apr. 21	-5.77	5.20	10.20

The cold snap during the melt season in 2016 that caused a secondary peak in AFDD resulted in discrepancies for the 2016 modelled water clear of ice date. Using the first AFDD peak (March 6^th^) to represent the maximum ice thickness, the model predicts no ice cover as of April 22^nd^ which is three days before the true water clear of ice date on April 25^th^ (Table 2). Using the actual maximum AFDD (April 13^th^) as the maximum ice thickness, the modelled water clear of ice date is after April 25^th^. As no in situ measurements could be collected during the melt process, we cannot compare the model results for ice decay to any measured data to investigate the 2016 season further.

### Estimating ice thickness using backscatter from RADARSAT-2

**Fig 5:**
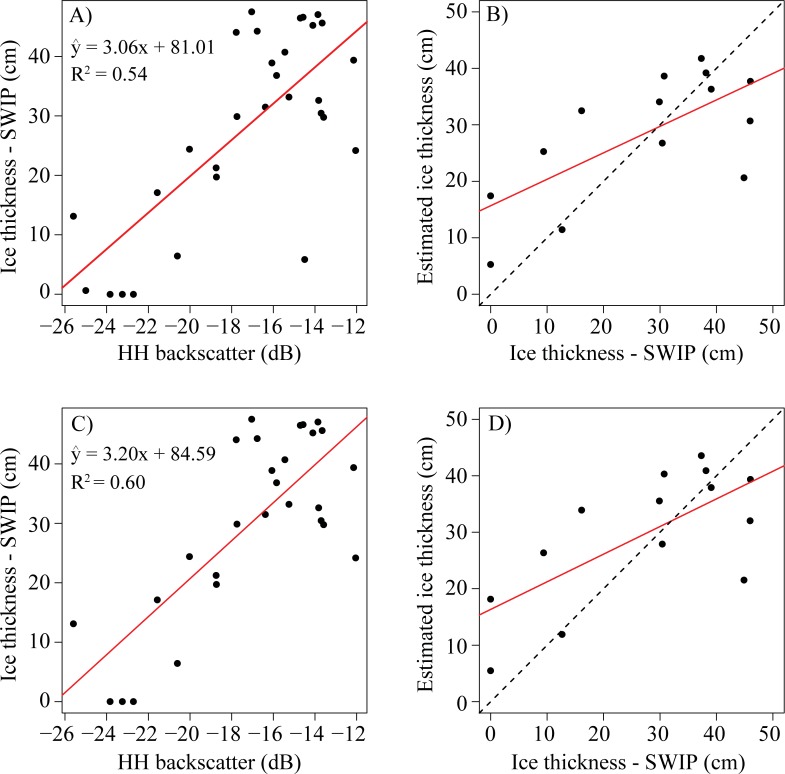
Results of developing a linear model for estimating ice thickness using HH backscatter. A) and B) (training and validation) show the result of using all data available, while C) and D) (training and validation) show the model result with the two identified outliers (December 10^th^ and 13^th^, 2016) removed. The dotted line represents a 1:1 relationship between estimated ice thickness and ice thickness measured from the SWIP. The red line in all plots displays the line of best fit.

The initial ice thickness model that is based on RADARSAT-2 backscatter and SWIP measurements is shown in Fig 5A. There is an increase of ice thickness by 3.1 cm for every 1 dB increase in backscatter response from the ice cover. The R^2^ value for the linear model was 0.54 (p-value < 0.01) (Fig 5A). The RMSE for the training data was 10.9 cm which is comparable to the RMSE for the validation data at 11.5 cm.

Two outliers were present in these data, these points are from the radar images acquired on December 10^th^ and December 13^th^, 2016, which have previously identified discrepancies with mean backscatter values of -25 and -14.5 dB respectively. These points are caused by unexpectedly low values due to thin skim ice (December 10^th^) and high values due to wave/possible rough ice surface during initial ice cover formation (December 13^th^) observed in shoreline observations. Once these data were removed, the ice thickness growth rate increased to a value of 3.2 cm per 1 dB increase and the R^2^ value increased to 0.60 (p < 0.01) (Fig 5C). The RMSE values were still comparable between the training and validation data, 9.5 and 11.7 cm, but the difference between values slightly increased compared to the model when all of these data were included (Fig 5). This model was selected over the initial model due to the increased R^2^ and comparable RMSE values and will be referred to as the ‘HH model’ from here on.

**Fig 6:**
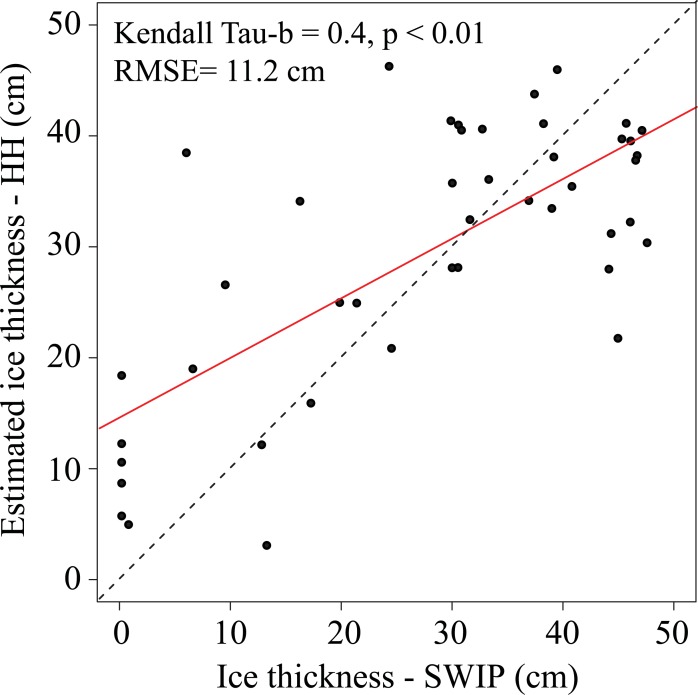
Estimated HH ice thickness and reported ice thickness from the SWIP. The red line represents the line of best fit and the dotted black line represents a 1:1 relationship.

When comparing all of the predicted values of ice thickness using HH backscatter compared to SWIP measurements, the model overestimates thin ice values and underestimates thicker ice values (Fig 6). On March 26^th^ and April 1^st^ 2017, near the beginning of melt for MacDonald Lake, there is a decrease in backscatter to values of -17 dB and -19.7 dB compared to values earlier in March that ranged from -12.1 dB to -16.1 dB. These decreases correspond with surface melt events captured in shoreline observations, however, ice thickness remains high (above 40 cm). Ice thickness reported by the SWIP on April 12^th^ is 24.4 cm, and while this date is also associated with a low backscatter value of -20 dB caused by surface melt (1.9° C), the HH model only under predicts ice thickness by 3.8 cm. Although backscatter normalisation techniques were used to limit backscatter variation between orbits, the differences in backscatter cause alternating increases and decreases of ice thickness not observable in the SWIP record, which can result in the noticeable over or underpredictions of ice thickness. On February 5^th^ the mean backscatter for MacDonald Lake was -13.8 dB (ascending orbit) and overestimated thickness by 7.8 cm, compared to the mean backscatter of -16.4 dB for the image captured the previous day (February 3^rd^, descending orbit) and the smaller difference of 0.8 cm. The use of mixed orbits has also been identified as an issue when attempting to detect ice phenology events [22,49].

**Fig 7:**
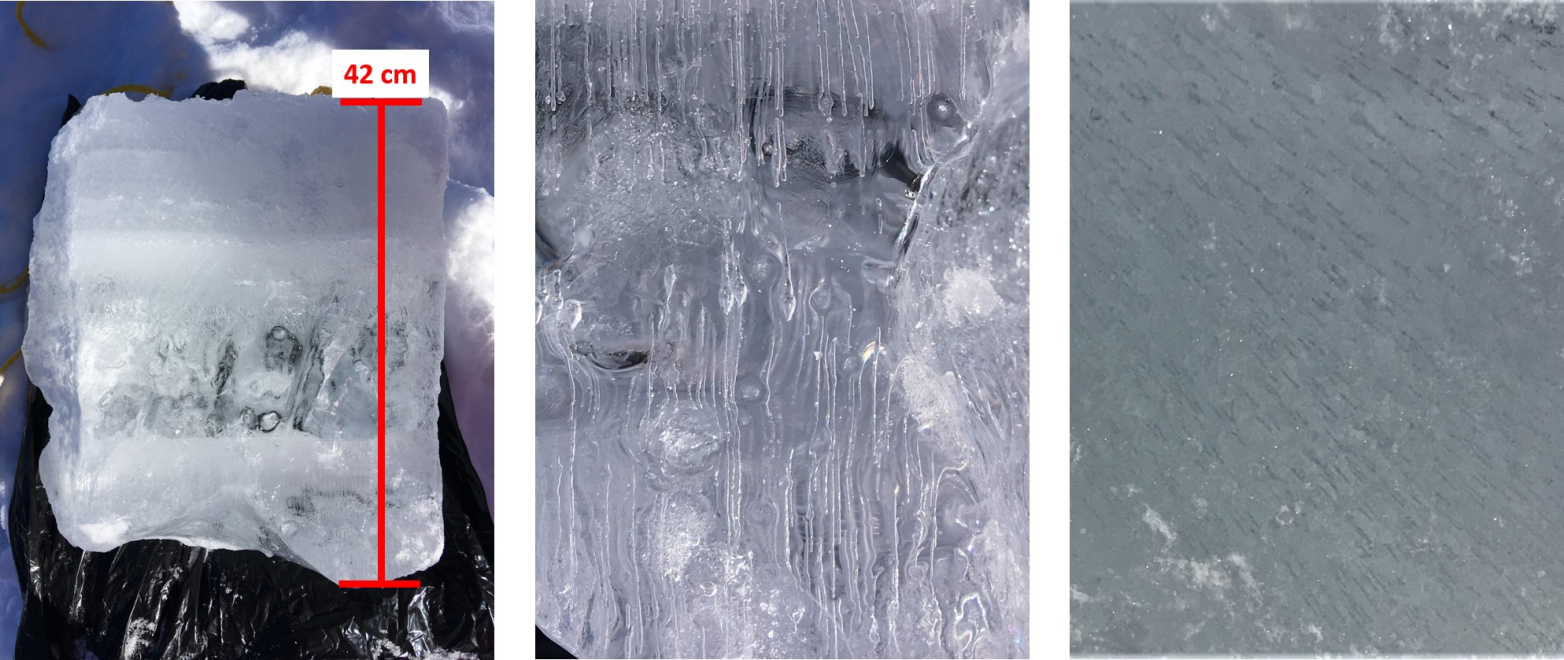
Ice block (42 cm) extracted from MacDonald Lake in Central Ontario. The stratification of the ice layer with top forming white ice (snow ice) and bottom growing clear ice is visible (left) with tubular bubbles present (centre) and zoom in on the bottom surface (right).

Although previous research suggests that backscatter should increase as ice thickens due to volume scattering caused by bubbles in the ice layers (eg. [50,51]), recently published research from Alaska and northern Manitoba suggests that surface scattering (single bounce) at the ice-water interface is the dominant scattering mechanism [52,53]. This may explain why backscatter does not accurately estimate ice thickness over the season, as the measured response may be due to changes in roughness at the ice-water interface, rather than increased scattering from bubbles as the ice grows [53]. As an example of variations in lake ice thickness, an extracted 42 cm block of ice obtained for MacDonald Lake on February 2, 2018, shows an uneven surface at the ice-water interface as well as tubular bubbles, both of which could contribute to roughness at the bottom of the ice cover (Fig 7). The exact mechanism for the ice-water interface roughening over time is yet unknown, however large-scale roughness is potentially related to the effect of snow cover on the ice and the associated variations in insulative properties resulting from snow redistribution, while finer scale variations in roughness may be the result of previously mentioned bubbles [53]. Also, the effect of thick snow ice on these scattering mechanisms, which forms when an increased mass of snow on the ice surface leads to the breaking and flooding of thin ice cover [54] or from refreeze during mid-winter melt events [23] that are common in mid-latitudes, requires further investigation.

### Comparison of ice thickness estimates between HH and temperature models

**Fig 8:**
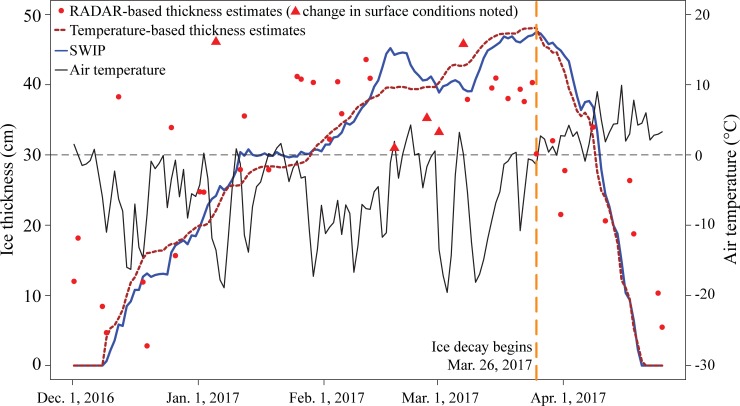
Comparison of the different ice measurement methods used as well as temperature (solid black line). The blue line represents the ice thickness measurements recorded by the SWIP, the dark brown line represents ice thickness estimated by temperature models, and the red points are the estimated ice thickness from the HH backscatter. The red triangles represent images that were acquired during a change in surface conditions causing errors. The vertical dotted orange line represents the transition between ice growth and ice decay on March 26^th^, 2017.

Ice thickness estimates for the 2016–2017 ice season from both the temperature and backscatter models are a good representation of the values that were obtained from the SWIP (Table 3, Fig 8). Index of agreement values support this with values of 0.93 when assessing temperature-estimated thickness compared to SWIP measurements and 0.67 for the backscatter-estimated thickness compared to the data from the SWIP. The backscatter model underestimates ice thickness when thickness is > ~40 cm, likely due to the previously mentioned influence of roughness at the ice-water interface. However, RADARSAT-2 could be useful in detecting ice thickness during the initial stages of thickening.

**Table 3:** Comparison of ice thickness from estimates and observations from the 2016–2017 ice season. Temperature-based estimates match the date of field observation, dates for the HH model and SWIP measurement are taken from the nearest date to the field observation date.

Date	Field Reported Ice Thickness ± Standard deviation (cm)	Degree Day Model (cm)	RADAR / SWIP Date	SWIP Measurement (cm)	HH Model (cm)
Jan. 20	28.78 ± 3.19	28.37	Jan. 19	29.87	27.89
Feb. 03	29.78 ± 2.06	33.02	Feb. 03	31.48	32.24
Feb. 10	33.05 ± 2.30	36.84	Feb. 12	37.32	43.58
Feb. 17	40.45 ± 3.58	39.57	Feb. 19	44.25	30.98
Feb. 21	44.38 ± 6.43	39.69	Feb. 19	44.25	30.98

Comparisons between these estimated data, SWIP measurements, and field observations (Table 3) show that the HH model does represent ice thickness well during periods of steady increase (January 20^th^ and February 3^rd^). However, the HH model is more affected by surface conditions than the temperature model causing over and under estimates. On February 19^th^, the HH model underestimated the ice thickness by 13.26 cm. This was due to a period of warmer temperatures that induced melt and increased water content in the snow; the increased water content would have absorbed the incoming beam from the RADARSAT-2 satellite resulting in the lower backscatter observed for the day, -16.8 dB. This can also be seen in Fig 8 where the values for February 27^th^ and March 2^nd^ (denoted by triangles) are underestimated compared to SWIP data and the estimated temperature-based ice thickness. The under estimation during this time is due to the change in surface conditions (visible pooled water on February 25^th^ and March 1^st^, and snowfall on February 27^th^), which was captured by shoreline observations. Additionally, on February 12^th^, the HH model overpredicts ice thickness (43.58 cm) compared to the closest field measurements for that day (33.05 cm) as a result of erroneously high backscatter (-12.84 dB) from the previously mentioned mix of ascending and descending orbits from the radar images.

### Application for long-term ice thickness

**Fig 9:**
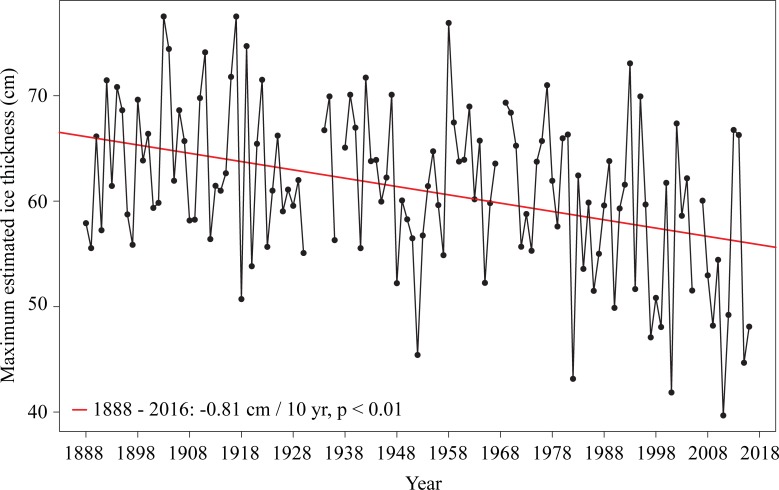
Long-term trend in ice thickness from 1888–2017 using the Adjusted and Homogenized Canadian Climate Data–Daily Temperature dataset.

Using the daily homogenized Canadian surface air temperature data (1888–2017), we developed a long-term estimate of maximum ice thickness for MacDonald Lake using the combined optimized AFDD model and ATDD model. There is a general trend toward lake ice thinning in Central Ontario at a rate of 0.81 cm per decade (p < 0.01) (Fig 9), however, the ice thickness estimates are highly variable with a large standard deviation of 7.78 cm (Fig 9). Additionally, no delayed model start date can be assessed for 1888–2007, as was done for 2008–2016. Comparing thickness estimates from 2008–2016 using the delayed model start based on freeze-up observations vs. thickness estimates using the model start date based purely on the AFDD, results in up to an 11.7 cm overestimation in the maximum ice thickness, adding some uncertainty to the < 2008 estimates. While significant thinning of the ice is displayed, these uncertainties should be kept in mind while interpreting the results.

Ice thickness trends in the Arctic (North Slope Alaska, 71.3° N) are more extreme with decreases in ice thickness modelled between 21 and 38 cm (1950–2011) and 18–22 cm (1991–2011) depending on snow cover [18]. Southern Finland (61.57° N) also showed significant trends for 4 of 11 lakes from 1961–2002, with certain sites, such as Päijänne, Tehi, showing a decrease in ice thickness of 3 cm per decade and significant trends to a lesser magnitude between 1912–2002, with sites such as Kuivajärv showing ice thinning by 1–1.1 cm [11]. Temperature trends help to explain why we observe greater trends in thinning ice for the Arctic and northern latitudes compared to the mid-latitudes, with the Arctic amplification of temperature trends resulting in the northern latitudes warming twice as fast as the lower latitudes in recent years [55,56]. Long-term trends (1900–2009) show upwards of 1–2°C increases in surface temperature for the Arctic, while trends of only 0.2 to 1°C are identified for the region of Southern/Central Ontario [56].

## Discussion and conclusion

The original Stefan’s model and the optimized AFDD model were found to accurately estimate ice thickness when compared to the SWIP and manual measurements. The optimized value for *β*_F_ in the first model, when a *γ* of 0.5 is used, was found to be 1.73. This value is within the range, 1.7–2.4, that has been noted in other literature for a lake with average snow cover [13,15].

Although the modified Stefan’s Eq (1) typically uses a constant γ value (0.5) and a *β*_F_ value dependent on surface conditions [12,15], the literature does occasionally use modified γ values. Models used to estimate sea ice thickness in the Arctic have modified both the *β*_F_ and γ values as done in this study. Lebedev [9], used a *β*_F_ value of 1.33 and a γ value of 0.58 for sea ice in the Arctic near Russia, while an equation developed for Button Bay near Churchill, Manitoba used a *β*_F_ value of 1.53 and a γ of 0.59 [9,10]. Although the *β*_F_ values for these studies are higher than what we found in Central Ontario for MacDonald Lake, the γ values are very similar (0.60), showing that the optimized AFDD model proposed for Central Ontario fits within the accepted literature. The use of this optimized model improved results compared to the modified Stefan’s Eq (1) (when a *γ* of 0.5 is used), resulting in lower RMSE when applied to the validation data, confirming that this method is appropriate for estimating ice thickness.

Ice thickness estimates were also accurately estimated during melt season when using the maximum ice thickness determined by the optimized AFDD model and an empirical cut-off of 90 ATDD to set the end of the ice season. The resulting estimates of ice thickness were strongly correlated with SWIP measurements with a Kendall Tau-b of 0.97 and a RMSE of 2.1 cm. The optimized AFDD model and the ATDD model can be combined in order to provide a complete record of thickness throughout the ice season, and the full season record was shown to have a high index of agreement (0.93) when compared with SWIP measurements. The combination of these models is the best choice when temperature data are available and phenology dates can be determined from satellite or other observation data. When no phenology data are available and only temperature data exists, the modified Stefan’s model (1) can still be used, with the caveat that the freeze timing based on AFDD alone may be too early in temperate latitudes. Future work could investigate the possibility of using a delay based on temperature during the ice-free season to more accurately represent the start of freeze in these latitudes. For larger regions where in situ temperature data are too sparse, the use of finer resolution gridded temperature data (e.g. ERA5 from ECMWF), or satellite-based temperature data (MODIS-LST), may be a viable option for driving the degree day models. The suggestion of using satellite-based temperature data supports the applicability of remote sensing to cover large spatial areas [57] and provide the appropriate coverage needed to produce accurate ice thickness measurements when in situ data are sparse or unavailable.

Although there is a higher amount of error for the model based on HH backscatter (R^2^ = 0.6, RMSE 11.7 cm), it does provide an alternative method to the degree-day models for isolated lakes where accurate temperature data may be unavailable, particularly for the initial growth period. On the other hand, the proposed model lacks some of the predictive strength observed in previous literature, e.g. R^2^ = 0.99 using C-band FMCW system in laboratory conditions [58]. The difference in predictive power between models is most likely due to the FMCW analysis using the increase in frequency at the ice/water compared to the constant frequency at the air/ice interface to determine thickness; the work being conducted in a laboratory with thinner ice cover of 24 cm compared to the lake ice in Central Ontario where thickness reached upwards of 47 cm; and the work being conducted on a smaller spatial scale (343 m^2^) compared to MacDonald Lake (~1.6 km^2^) [24,58]. Research in Central Ontario is limited by the temporal availability of SAR images, which restricts the analysis to only focusing on HH backscatter as a predictor for thickness and requires the use of both ascending and descending orbits, which can result in errors in the HH model. Additionally, in the temperate latitudes where mid-winter thaws are experienced, the changes in water content in the on-ice snow cover and occasional pooling on the ice caused by melting or precipitation events also reduces the accuracy of the HH model. Tests of a linear regression (note: no training or validation set was used due to the limited number of data points) during the early stages of ice thickness (prior to 40 cm, December 2^nd^, 2016 to February 13^th^, 2017), found a significant relationship with a high R^2^ of 0.66 and a lower RMSE compared to the model developed for the full ice season when all data points are used (6.8 cm vs. 11.1 cm). This shows that HH backscatter may have the most applicability during the early stages of ice growth.

In order to improve the results of the HH model, further investigation into how temperate latitude lake ice affects HH backscatter is needed. Additionally, there is a need for a better understanding on how the initial formation of snow ice impacts backscatter. Snow ice has also been previously reported as being a large contributor to scattering in temperate latitude lake ice due to the large number of bubbles found in this layer [50,59]. Snow cover also plays an important role in regulating ice thickness as increases to the snow mass during the formation of ice can have an impact on the rate of ice growth as well as the type of ice that forms [60–63]. Additionally, if there is snow redistribution on the surface of the lake this may affect spatial differences in the thickness of ice across the lake [53]. In mid-latitudes slushing events caused by mid-winter precipitation events and warmer temperatures can also lead to increases in ice thickness through snow ice growth [23].

Overall, lake ice thickness is more accurately estimated in Central Ontario using temperature models compared to models based on HH backscatter. Both the modified Stefan’s equation and optimized AFDD model were able to accurately predict ice thickness based on measured SWIP data. However, the optimized AFDD model does require in situ or satellite observations to capture the start of freeze onset to increase accuracy. The equations are presented as a simple method that can be used to estimate ice thickness for areas where nearby temperature data are available. This could be quite beneficial for communities that rely on ice thickness for tourism and recreation to supplement their manual measurements, or who may be interested historical thickness trends for their area (with the caveat that thickness maybe overestimated without including the freeze-up timing). Furthermore, the identified cut-off of 90 ATDD for water clear of ice could be used to improve automated methods of identifying ice phenology from remote sensing images by providing an estimate for the end of the ice season. RMSE values from the optimized AFDD model and ATDD model were 2.3 cm during ice growth and 2.1 cm during ice decay with correlation statistics greater than 0.9 for both ice growth and decay. The combined AFDD and ATDD model was also used to show that ice thickness for MacDonald Lake, and likely all small/medium sized lakes in Central Ontario, has been thinning since temperature records began there in 1888.

This work also raises the question of whether the observed HH backscatter increase is a result of single-bounce scattering from the roughening of the ice-water interface over time, as determined for northern lakes [53], rather than a direct relationship between volume scattering and thickening ice. This question could be addressed by studying the structure of temperate lake ice, both within the ice layer and at the ice-water interface, and the effect of surface scattering, particularly during formation when manual samples cannot be extracted. Developing an algorithm from SAR data would allow for the estimation of ice thickness over a large area such as Central Ontario that would be unaffected by limitations such as spatial variations in temperature and allow for better monitoring of ice thickness.

## Supporting information

S1 File SupplementaryData_2015_2017. The supplementary data file includes metadata information about the location of the weather station at MacDonald Lake, full temperature data from the weather station for 2015/2016 and 2016/2017 ice seasons, and SWIP recorded ice thickness data for 2016/2017.
(XLSX)
